# Cross-Cultural Validation of Quebec User Satisfaction with Assistive Technology 2.0 for Spanish Population (QUEST-2.0 ES)

**DOI:** 10.3390/ijerph19159349

**Published:** 2022-07-30

**Authors:** Joao Guerreiro, Estíbaliz Jiménez-Arberas, Patricia Porto Trillo, Olalla Vilar Figueira, Pedro Saénz-López Buñuel, Sandra Pais, José Tierra Orta, Thais Pousada García

**Affiliations:** 1 Independent Researcher, 8005-160 Faro, Portugal; jqguerreiro@gmail.com; 2 Faculty Padre Ossó, University of Oviedo, 33008 Oviedo, Spain; estibaliz@facultadpadreosso.es; 3 Galician Confederation of People with Disabilities (COGAMI), 15704 Santiago de Compostela, Spain; patriciaportotrillo@gmail.com (P.P.T.); cegadi@cogami.es (O.V.F.); 4 EMOTION Research Group, University of Huelva, 21071 Huelva, Spain; psaenz@uhu.es; 5 Faculty of Medicine and Biomedical Sciences, Universidade do Algarve, 8005-135 Faro, Portugal; spais@ualg.pt; 6 Comprehensive Health Research Centre (CHRC), 1150-082 Lisboa, Portugal; 7 Department of Integrated Didactics, University of Huelva, 21071 Huelva, Spain; jtierra@uhu.es; 8 CITIC, TALIONIS Research Group, University of A Coruña, 15670 A Coruña, Spain

**Keywords:** outcome measures, assistive technology (AT), validation, Spanish, satisfaction

## Abstract

Background: Assistive technologies (ATs) are resources to promote the independence and participation of people with a disability. The use of standardized tools, based on outcome measures, is essential for guaranteeing high-quality rates. The Quebec User Evaluation of Satisfaction with AT–2.0 (QUEST) is a scale to assess the satisfaction of people using any AT. Objectives: To translate and culturally validate the QUEST–2.0 for the Spanish population (QUEST 2.0-ES). Methods: A validation cross-design and descriptive study. The test–retest reliability, validity, and internal consistency of QUEST 2.0-ES were studied. It was divided into two phases: Sample 1 was formed by 26 persons; in sample 2, 30 persons participated. The conditions included neurological conditions, amputations, rare diseases, and deafness. Results: Thirty-five men and 21 women participated in total. The majority of AT used were those for mobility. QUEST 2.0-ES analysis showed internal consistency values between the test (α = 0.87) and retest versions (α = 0.89). The internal consistency was high for AT (test, α = 0.83; retest, α = 0.87) and Service (test, α = 0.80; retest, α = 0.80). The temporal reliability (1–2 weeks) for test–retest was 0.88. Conclusion: QUEST 2.0-ES showed good psychometric properties in terms of validity and test–retest reliability, and it is a good tool to assess the user’s satisfaction with ATs and services.

## 1. Introduction

Assistive technology (AT) refers to different devices especially developed or assembled as solutions for people with a disability or impairment, and they include several categories such as mobility, sensory assistance, communication, personal care, and recreation [1,2]. AT can facilitate participation indirectly (treatment or therapy) or directly (physical assistance) by enhancing an individual’s mobility and activities of daily living; and other vital areas, such as education, work, or leisure [2,3]. Physical rehabilitation professionals require expertise to select AT, and they must consider factors related to the person, the AT itself, and the environment where it will be used [4]. Therefore, the prescription of an AT should be a systematic process, with a multidisciplinary team at the center, focusing on the individual [5], and incorporating an assessment of their needs, priorities, and skills. Evaluation instruments, including questionnaires or scales, provide high-quality assessment standards for AT services, and assist in measuring outcomes, which assist in determining whether the AT fulfills its technical requirements and its level of efficiency [6]. The use of standardized methods is also critical for the success of AT, including the follow-up process and adherence to AT [6,7,8], and individual expectations, perspectives, attitudes, and values, in order to reduce abandonment of its use [9].

The Quebec User Evaluation Satisfaction with Assistive Technology–2.0 (QUEST–2.0) is a self-administered instrument that was developed to measure satisfaction with a wide range of ATs and related services. Its stability and reproducibility are well described [10,11,12,13,14]. QUEST–2.0 is integrated using two subscales: (1) devices and (2) services, and a score is created for each, and summed for a total score. So, the user of AT is asked to rate his/her satisfaction with respect to different aspects regarding the AT and the service provided, using a five-point scale ranging from 1 (not satisfied) to 5 (very satisfied). The device subscale (ATS) questions concern the dimension, weight, adjustment, safety, durability, simplicity of use, comfort, and effectiveness (eight questions) of it, and the subscale for services (SV) assesses the provided assistance for the device, namely: service delivery, repairs, professional services, and follow-up services (four questions). A score for AT (ATS), a score for service (SV), and a final total score (TS) are obtained using the sum of both sub-scales [10,12,14]. QUEST–2.0 has already been translated and validated into different languages, and the versions present good internal consistency (0.79–0.90) and test–retest reliability (0.64–0.97) [15,16,17,18,19,20,21]. A recently published study demonstrates the validity and reliability of QUEST-IT 2.0 for an Italian population using mobility assistive devices [22] and for athletes who use a sports wheelchair [23].

Spain has an estimation of 45 million inhabitants, and it is estimated that there are 1.5 million potential users of assistive technology (3.19%), but the majority do not have sufficient information regarding the device suited to their needs, nor its availability [24]. The last available survey concerning the usage of assistive devices in Spain, although not recent, does indicate that only 38% of men and 41% of women with a disability limiting their activities of daily living, used any AT [25]. According to the usage rates of the different types of ATs in Spain, the survey found that devices for mobility are the most commonly used (61.1% for those who used any AT), followed by devices for personal care (32%), and orthoses and prostheses (28.3%). The least utilized ATs were those for leisure (8.5%) [24].

In addition, as there are only two instruments validated for the region: the Psychosocial Impact of Assistive Device Scale (PIADS) [26], and the Matching Person & Technology (MPT) model [27], the use and implementation of outcome measures by professionals in the Spanish healthcare system is scarcely utilized [28].

Considering the applicability of this instrument and its importance in physical rehabilitation and AT outcome measures, the objective of the study was to translate and culturally validate the Quebec User Evaluation of Satisfaction with Assistive Technology–2.0 for the Spanish population (QUEST 2.0-ES). The psychometric properties studied were test–retest reliability, validity, and internal consistency. As a secondary objective, the satisfaction of the participants with their AT used and related services was assessed.

## 2. Materials and Methods

The process of cross-cultural validation for Spain was carried out in two phases and included translation, synthesis of the translation, back translation, an expert committee, and a trial of the prefinal version, as recommended by Beaton [29]. Before commencing, permission and authorization for the translation and cross-cultural validation were obtained from the authors of the original questionnaire.

### 2.1. Procedure

The first phase included translation, back translation, an expert committee, and the prefinal version. The translation was performed by two translators, one English native speaker with knowledge of the Spanish language, and another Spanish native speaker with a degree in Physical Activity, and who was fluent in English. Both translators worked separately and were blind to each other’s translation until the synthesis of the translations was carried out. Back translation was carried out, blind to the original and previous versions, by a Spanish native speaker with extensive experience with the English language, who holds a degree in Prosthetics and Orthotics.

An expert committee was formed and was composed of an academic investigator with experience in questionnaire application and cross-cultural validation, as well as a physician. Each worked separately and completed a report regarding the semantic, idiomatic, experimental, and conceptual equivalence of the translated version in comparison to the original, translation, and back translation documents. A prefinal version was developed, which was applied to a sample made up of 26 individuals, who volunteered to participate in the test–retest process (1 to 2 weeks), from associations and adaptive sports clubs in the Spanish regions of Badajoz and Caceres. 

Following phase one, different professionals in the field of rehabilitation considered the original translation of QUEST 2.0-ES to be lacking, as the first version did not correctly and completely reflect the terms from the original, nor was it sufficiently adapted to the cultural background of the population using AT. Therefore, a second phase was carried out to improve the conceptualization of the items, and to integrate language that was more applicable to the field of rehabilitation, according to the original instrument in English. This included a review and cultural adaptation of the questionnaire, the formation of an expert committee, and an improved prefinal version. The main researcher, along with a second researcher who specialized in occupational therapy, reviewed the terminology and cultural applicability to the Spanish population, of the first version. This was carried out in conjunction with the author of the original questionnaire.

In this second phase, the expert committee was made up of four Occupational Therapy (OT) professionals, with expertise in the prescription of and intervention with AT, as well as in the application of scales for outcome measures. These professionals carried out a review of the original questionnaire in Spanish, suggesting pertinent changes according to the guidelines indicated by Beaton et al. [29]. The individual reports were filtered and consolidated into a single and final document that formed the instrument to be validated. This second version in Spanish was applied to the test–retest procedure with a sample of 30 participants from the regions of Galicia, Asturias, and Castilla León. This was carried out with the support of Occupational Therapists, working with associations for people with disabilities, over a period of 2 weeks (test–retest).

In both phases, the administration of the assessment tool was conducted as an interview between the professional and the AT user. All participants in the complete study (first and second phase) completed the informed consent forms before the study.

### 2.2. Characteristics of Sample and Variables

In the first phase, the sample was made up of 26 individuals, while in the second phase, the sample consisted of 30 individuals. In both phases, participants met the following inclusion criteria: adults (older than 18), with physical and/or sensorial disabilities (to a moderate or high degree), having and frequently using any AT, and possessing a good understanding of the Spanish language, along with sufficient cognitive skills to understand the questionnaire and the research procedure.

The main variable of the study was the self-perception of satisfaction with the assistive device used, in relation to the subdimensions of the AT and related services (according to the dimensions included in the QUEST–2.0). The other variables were sociodemographic characteristics: age, gender, diagnosis (or functional status), place of residence, AT used, and length of time using the assistive device.

All gathered data were previously codified to maintain the confidentiality of the participants, and the research group used REDCap (Research Electronic Data Capture) for data collection. REDCap is a secure, web-based software platform designed to support data capture for research studies [30].

### 2.3. Data Analysis

A descriptive analysis of the variables considered was carried out, with the determination of the mean and standard deviation, as well as the frequency and percentage of the numerical and categorical variables, respectively.

The study of internal consistency for QUEST 2.0-ES was carried out with Cronbach’s alpha test, while the test–retest reliability of both the global score and the individual items was analyzed through the Spearmen’s Rho, and content validity was analyzed using the Intraclass Correlation Coefficient.

The precision criterion of α = 0.05 was established for hypothesis testing, to validate the level of significance to implement the statistical test, and to determine the presence of significant correlations between variables. The statistical program used was Statistical Package for Social Sciences version 24 (SPSS V.24), (IBM SPSS Statistics for Windows, Version 24.0. IBM Corp., Armonk, NY, USA) SPSS^®^ v.24.

Cronbach’s alpha (α) values range from 0 to 1 and can be grouped as unacceptable (<0.5), poor (0.5–0.6), acceptable (0.6–0.7), good (0.7–0.9), and very good (0.9–1) [31]. Spearman’s Rho can range from −1 to 1 (perfect correlation), with zero identifying no correlation between variables [32,33]. The correlation can be also considered as weak (0.1–0.3), moderate (0.4–0.6), and strong (0.7–0.9). Intraclass correlation coefficient values can indicate poor reliability (<0.5), moderate reliability (0.5–0.75), good reliability (0.75–0.9), and excellent reliability (0.9–1) [33,34].

### 2.4. Ethical Concerns

This study meets the current ethical standards that regulate research on humans, including the Helsinki Declaration, the Oviedo Convention on Human Rights and Biomedicine, the Regulation (EU) 2016/679 of the European Parliament, and of the Council of 27 April 2016 on the protection of natural persons with regard to the processing of personal data. Phase one of the study was approved by the ethical committee of the University of Huelva (3 October 2013). Phase two of the study has the approval of the Ethics Committee of the University of A Coruña (December 2020) with the reference number: 2020/2026.

All participants gave informed consent after reading the information document of the study.

## 3. Results

### 3.1. Descriptive Statistics

In this section, results are presented according to the first phase (sample 1) or the second phase (sample 2) in Table 1.

Sample 1: A total of 26 individuals (30.8% ♀; 65.4% ♂; 3.8% unknown) were successfully selected and completed the test–retest versions. The total sample was divided according to the etiology of the injury, into spinal cord injury (38.46%), neuromuscular disorders (3.85%), lower limb amputation (7.69%), brain injury (7.69%), and other/not specified (42.31%). Sample 1 subjects only use devices for mobility, and are organized into manual wheelchair (*n* = 7), prosthetic devices (*n* = 1), powered wheelchair (*n* = 6), walkers (*n* = 1), walking canes (*n* = 2), ankle–foot-orthosis (*n* = 1), and other types not specified by the users (*n* = 8).

Sample 2: A total of 30 individuals participated in the second phase, where (62.1% ♂; 37.9% ♀). Similar to phase 1, the diagnosis was divided into the etiology of the disability; namely, deafness (30%), SCI (23.3%), stroke (23.3%), neuromuscular disorders (NMD)(16.7%), traumatic brain injury (3.3%), and visual impairment (3.3%). Regarding the type of AT used by the participants, these have been grouped into the following categories: mobility (46.7%), hearing aids (30%), feeding (6.7%), read/write (6.7%), grooming (3.3%), aid for domestic activities (3.3%), and dressing (3.3%). The majority used their AT all day (70%), and none used the device sporadically (once per week). The devices were used more frequently both indoors and outdoors (66.7%), with only five individuals using the AT solely indoors, and a further five individuals using it only outdoors.

In sample 1, the result from the application of QUEST–2.0 for the test version TS is 3.87 ± 0.81 (ATS = 3.95 ± 0.82; SV = 3.69 ± 0.98). In the retest, the values are similar in TS (3.77 ± 0.82) (ATS = 3.92 ± 0.83; SV = 3.47 ± 0.99). The results in sample 2 are comparable, with a mean QUEST result for the test version TS of (3.93 ± 0.74), and SV (3.65 ± 1.24), while the values for ATS (4.08 ± 0.59) are higher than those obtained in sample 1. No significant variance was observed with respect to the results obtained for retest in TS (3.98 ± 0.77), ATS (4.16 ± 0.63), and SV (3.63 ± 1.2) (Table 2). In sample 1, the three most important items identified in ATS, by the study participants in both the test and retest were “safety” (22.1%), “comfort” (16.9%), and “effectiveness” (13%). In sample 2, the three most important items selected were “easy to use” (22.58%), “weight” (14.89%), and “comfort” (10.64%) (Table 3).

### 3.2. Psychometrics

For cross-cultural validation, psychometric statistical tests of reliability in alpha analysis for internal consistency (α), slip file to test–retest reliability (ρ), and the performance of intra-class correlation coefficient, were used (Table 4).

The QUEST 2.0-ES analysis of sample 1 shows internal consistency TS values between the test (α = 0.87) and retest versions (α = 0.88). Internal consistency is high for AT (test, α = 0.88; retest, α = 0.89) and SV (test, α = 0.65; retest, α = 0.63). Temporal reliability (1 to 2 weeks) for test–retest in TS is 0.97, with similar values for AT (ρ = 0.98) and SV (ρ = 0.89). An ICC (95%) of 0.94 (0.91–0.97), 0.81 (0.68–0.91), 0.94 (0.89–0.97) for ATS, SV, and TS, respectively, was found. 

The QUEST 2.0-ES analysis for sample 2 shows internal consistency. TS values between test (α = 0.89) and retest versions (α = 0.91). Internal consistency is high for ATS (test, α = 0.78; retest, α = 0.83) and SV (test, α = 0.90; retest, α = 0.91). Temporal reliability (1 to 2 weeks) for test–retest in TS is 0.78, with similar values for ATS (ρ = 0.74) and SV (ρ = 0.75). An ICC (95%) of 0,87 (0.79–0.93) for ATS, 0.92 (0.86–0.95) for SV, and 0.93 (0.89–0.96) for TS, was found. 

The QUEST 2.0-ES analysis using the results of both samples (Table 5) shows internal consistency. TS values between test (α = 0.87) and retest versions (α = 0.89). Internal consistency is high for ATS (test, α = 0.83; retest, α = 0.87) and SV (test, α = 0.80; retest, α = 0.80).

The temporal reliability (1 to 2 weeks) for test–retest in TS is 0.88, with similar values for ATS (ρ = 0.90) and SV (ρ = 0.81). An ICC (95%) of 0.92 (0.88–0.95) for ATS, 0.87 (0.81–0.92) for SV, and 0.93 (0.90–0.96) for TS, was found. 

## 4. Discussion

As previously mentioned, assistive technology are devices, tools, and resources for increasing the independence in the activities of daily living of people with disabilities. However, these products on their own and without prior education and evaluation, will oftentimes not meet the expectation of the individual, if their unique characteristics, activity, and environment are not taken into account. Outcome measures are tools that support the work of professionals in the process of prescribing, providing, and conducting the follow-up after intervention with any AT [8,35].

QUEST 2.0 is one such instrument that allows the satisfaction of the user (person with a disability) with her/his assistive device and the process of prescription (service) to be evaluated [14]. 

The present study aimed to translate, adapt, and validate the Quebec User Satisfaction with assistive technology 2.0 in the Spanish population.

The validation process of QUEST 2.0-ES demonstrated high internal consistency values in the total score between the test (α = 0.87–0.89) and retest versions (α = 0.88–0.91), both in sample 1 and sample 2. The internal consistency was high for ATS (test, α = 0.88–0.78; retest, α = 0.89–0.83) and SV (test, α = 0.65–0.90; retest, α = 0.63–0.91). When compared to the Portuguese version, the Spanish version presents similar values in internal consistency (α = 0.79) for the total score and the subscale of assistive technology (α = 0,80) while only the Service subscale (α = 0.81) was found to be lower [21]. However, in the joint analysis of both samples, it was found that the internal consistency values for test and retest ranged from 0.83 to 0.89. The Italian (α = 0.74) and Dutch versions (α = 0.73 and 0.85) shows similar values with respect to the internal consistency [15,22].

Temporal reliability between 1 to 2 weeks for test–retest had a total score of 0.78–0.97, with similar values for the Assistive Technology Subscale (ρ = 0.74–0.98) and Service (ρ = 0.75–0.89). Compared to the Portuguese version, the test–retest reliability (7 to 11 days) presented similar and good values for a Total Score of ρ = 0.93, and for Assistive Technology subscale (ρ = 0.89) and Service (ρ = 0.94) [21]. Using the joint analysis, it was found that the temporal reliability values were higher and ranged from 0.88 to 0.90.

Compared to the Taiwanese version of QUEST–2.0, it was found that the Cronbach’s alpha of the ATS, SV, and TS were 0.87, 0.84, and 0.90, respectively, indicating a high internal consistency. With regard to the test–retest reliability, the ICCs (95% CI) for ATS and SV, and TS were 0.90, 0.97 and 0.95, respectively, indicating an excellent score agreement [17]. The values for internal consistency were similar to those in the Spanish version. The ICC values were also similar to the Taiwanese version, with only SV being lower. The values were similar to the values of the Spanish version for Colombia, which presented high values in ATS (α = 0.89), SV (α = 0.82), and TS (0.91) [18]; and with the recent results of the Korean version [19], with ATS (α = 0.88; ρ = 0.64) and SV (α = 0.92; ρ = 0.66); and the Brazilian versions [20] with an internal consistency of ATS (0.86), SV (0.72), and TS (0.83). 

Recently, a version of QUEST 2.0 for the Italian population was validated and showed good reliability. In comparison, the results of the Spanish version were marginally higher with regard to the Cronbach’s alpha values (for ATS, SV, and TS); however, in contrast, the ICC values were higher in the Italian version [36]. Recently, this tool has also shown good psychometric properties and applicability to determine the satisfaction of athletes using their sports wheelchairs [23]. 

Considering the results between sample 1, sample 2, and the joint analysis, it was found that by enlarging the sample and by using subjects requiring different assistive technologies, the results were higher and closer to the different validated versions of QUEST–2.0.

Although there is a high number of customized (handmade) and standardized assistive devices, there is a growing movement for low-cost 3D-printed and CAD-CAM assistive devices. The application of instruments to validate the adaptation, efficiency, and effectiveness of these devices is still low [28,37,38], and there is limited research in which the QUEST was applied to novel and low-cost AT [39,40]. Therefore, it is important to consider the use of tools such as QUEST 2.0-ES to improve the outcomes of the design and use of low-cost assistive technologies, and to determine their potential effectiveness and efficiency.

### Study Limitations

The research was conducted in two different contexts and time periods, utilizing two samples, each with specific diagnoses and assistive devices. This has allowed for an enrichment of the results obtained, and a consolidation of the psychometric properties. However as the data derived from two specific contexts, and its generalization could be complex, these results should be interpreted cautiously. In addition, the cross-cultural validation guidelines used in this research [29] recommends 30 to 40 subjects for the pre-final version of the scale validation. In this study, 56 subjects fulfilled the test–retest versions in the joint analysis of the samples, in accordance with the used guidelines. However, the study population (AT users) is very specific, and this narrows the chance of contacting more study subjects. 

Phase two of the research was carried out during the COVID-19 pandemic, which may have influenced the participants’ daily activities and willingness to participate.

We consider that the development of a pre-test version with the scale, and a discussion group with AT users before the development of the test–retest process would have enriched the QUEST-ES validity. However, due to the difficulty in contacting AT users and in matching the inclusion criteria; the cooperation of non-profit associations and health staff, who are crucial for establishing contact with AT users; the need for delivering the scales on site to carefully explain the nature of the study and the importance of repeating it a second time (retest); and the lack of funding, which made it impossible to cover a wider area of Spanish territory; we did not use a pre-test group. Spain has an estimated of 45 million inhabitants, and it is estimated that there are 1.5 million potential users of assistive technology (3.19%). Considering the Spanish population distribution according to autonomic regions, and the estimation of AT potential users in each region, it is estimated that the areas where the study was developed present some of the lowest numbers of potential AT users—Extremadura (33,500), Galicia (85,000), Asturias (32,000), and Castilla Leon (75,400).

In the second phase, and due to the geographical dispersion of the participants and the confinement derived from the COVID-19 pandemic, we did not have the opportunity to develop a discussion group with AT users from sample 2. We recognize that this process would have improved the research.

The availability of the validated QUEST-ES for the Spanish context is an opportunity to continue the research in this field. Professionals and researchers have a new tool to apply the outcome measures to the intervention with assistive technology, and this application will contribute to increase the results in this field, generating new findings and contributing to the data analysis that was commenced in the present study.

## 5. Conclusions

The research presented has been developed in Spain in two different phases and periods, and offers a good perspective regarding the satisfaction of the AT users with their devices and related services.

- The QUEST 2.0-ES version has shown good psychometric properties in terms of validity and test–retest reliability, and it is a good tool for assessing the user’s satisfaction with assistive technology and the services linked to it.
- In general, the user’s satisfaction with the AT assessed is high or very high in both samples, and the score regarding the device itself is higher in comparison to the service for AT Scale. The items most important to the participants with regard to satisfaction are: “easy to use”, “weight”, and “safety”.
- The assistive devices assessed were grouped into seven types, with those for grooming and domestic activities being the devices with the highest scores.

## Figures and Tables

**Table 1:** Descriptive results from sample 1 and sample 2.

Variables		Sample 1		Sample 2	
		N	%	N	%
Gender	Unknown	1	3.8		
	Men	17	65.4	18	62.07
	Women	8	30.8	11	37.93
Type of Diagnosis	Spinal Cord Injury	10	38.46	7	23.33
	Neuromuscular Disorders	1	3.85	5	16.67
	Deafness			9	30
	Amputation (Lower limb)	2	7.69		
	Brain Injury	2	7.69	7	23.33
	Traumatic Brain Injury			1	3.33
	Blindness/Low Vision			1	3.33
	Other/Not Specified	11	42.31		
Type of AT	Mobility	26	100	14	46.67
	Hearing Aids			9	30
	Grooming			1	3.33
	Dressing			1	3.33
	Feeding			2	6.67
	Read/Write			2	6.67
	Domestic Tasks			1	3.33
Range age	Younger than 25	1	3.8	4	13.33
	26–35	6	23.1	3	10
	36–45	10	38.5	7	23.33
	46–55	3	11.5	4	13.33
	Older than 56	5	19.2	12	40

**Table 2:** Results from the application of QUEST 2.0-ES per item of the scale.

	Sample 1 (*n* = 26)				Sample 2 (*n* = 32)			
	Test		Retest		Test		Retest	
	Mean	SD	Mean	SD	Mean	SD	Mean	SD
Dimensions	4.23	0.82	3.85	1.01	4.07	1.05	4.07	1.01
Weight	3.69	1.16	3.46	1.30	4.20	1.00	4.20	0.96
Adjustments	3.73	1.19	4.00	1.06	3.97	0.89	4.07	0.87
Safety	3.69	1.29	3.88	1.18	4.20	0.81	4.33	0.71
Durability	3.92	1.26	4.00	1.10	4.00	0.95	4.13	1.01
Easy to use	4.15	1.01	4.15	0.97	4.40	0.77	4.53	0.57
Comfort	3.81	1.30	3.77	1.24	3.80	1.06	3.93	1.17
Effectiveness	4.35	0.85	4.23	0.82	3.97	1.00	4.00	1.08
Service delivery	3.58	1.47	3.42	1.36	4.10	1.12	4.10	1.09
Repairs/servicing	3.35	1.47	3.35	1.26	3.40	1.43	3.37	1.47
Professional service	3.69	1.41	3.35	1.44	3.63	1.45	3.57	1.43
Follow-up services	3.46	1.75	3.23	1.73	3.47	1.57	3.47	1.38
Assistive technology score	3.95	0.82	3.92	0.83	4.075	0.591	4.158	0.63
Services score	3.69	0.98	3.47	0.99	3.65	1.24	3.63	1.20
Total score	3.87	0.81	3.77	0.82	3.93	0.74	3.98	0.77

**Table 3:** QUEST 2.0-ES item satisfaction percentage distribution.

	Sample 1		Sample 2	
	Test	Retest	Test	Retest
Dimensions	6.5%	8.1%	7.53%	10.64%
Weight	7.8%	6.8%	13.98%	14.89%
Adjustments	7.8%	5.4%	8.60%	6.38%
Safety	22.1%	21.6%	7.53%	7.45%
Durability	11.7%	9.5%	5.38%	11.70%
Easy to use	9.1%	10.8%	22.58%	22.34%
Comfort	16.9%	20.3%	9.68%	10.64%
Effectiveness	13.0%	13.5%	12.90%	9.57%
Service delivery			3.23%	2.13%
Repairs/servicing	2.6%	2.7%	5.38%	2.13%
Professional service			1.08%	
Follow-up services	2.6%	1.4%	2.15%	2.13%

**Table 4:** Psychometric analysis in sample 1 and sample 2.

Items	Sample 1				Sample 2			
	Cronbach Alpha		Spearman’s Rho	ICC (95%)	Cronbach Alpha		Spearman’s Rho	ICC (95%)
	Test	Retest			Test	Retest		
ATS Score	0.88	0.89	0.98	0.94 (0.91–0.97)	0.78	0.83	0.74	0.87 (0.79–0.93)
Services score	0.65	0.63	0.89	0.81 (0.68–0.91)	0.90	0.91	0.75	0.92 (0.86–0.95)
Total score	0.87	0.88	0.97	0.94 (0.89–0.97)	0.89	0.91	0.78	0.93 (0.89–0.96)

**Table 5:** Psychometric joint analysis of sample 1 and sample 2.

Items	Joint Analysis of Sample 1 and Sample 2			
	Cronbach Alpha		Spearman’s Rho	ICC (95%)
	Test	Retest		
ATS	0.83	0.87	0.90	0.92 (0.88–0.95)
SV	0.80	0.80	0.81	0.87 (0.81–0.92)
TS	0.87	0.89	0.88	0.93 (0.90–0.96)

## Data Availability

No new data were created or analyzed in this study. Data sharing is not applicable to this article.
